# Butylphthalide Combined With Conventional Treatment Attenuates MMP-9 Levels and Increases VEGF Levels in Patients With Stroke: A Prospective Cohort Study

**DOI:** 10.3389/fneur.2021.686199

**Published:** 2021-12-20

**Authors:** Yingqiong Xiong, Juanjuan Liu, Yang Xu, Shu Xie, Xinhua Zhou, Shaomin Cheng

**Affiliations:** ^1^Department of Neurology, the First Affiliated Hospital of Nanchang University, Nanchang, China; ^2^Department of Neurology, Jiangxi People's Hospital, Nanchang, China; ^3^School of Chinese Medicine, Jiangxi University of Traditional Chinese Medicine, Nanchang, China

**Keywords:** stroke, prognosis, vascular endothelial growth factor, matrix metalloproteinase 9, butylphthalide

## Abstract

**Background and Purpose:** Butylphtalide increases the vascular endothelial growth factor (VEGF) and decreases matrix metalloproteinase (MMP)-9 in animal models of stroke and might be of use in the management of stroke. To explore whether butylphthalide combined with conventional treatment can change the levels of MMP-9 and VEGF and the National Institutes of Health Stroke Scale (NIHSS) scores of patients with stroke.

**Methods:** This was a prospective cohort study involving inpatients admitted to the Jiangxi Provincial People's Hospital (January–June 2019) due to acute cerebral infarction. The patients received conventional treatments with or without butylphthalide. The changes in the NIHSS scores were compared between groups. Plasma MMP-9 and VEGF were measured by enzyme-linked immunosorbent assay.

**Results:** A total of 24 patients were included in the conventional treatment group and 46 in the butylphthalide group. The butylphthalide group showed lower MMP-9 (130 ± 59 vs. 188 ± 65, *p* = 0.001) and higher VEGF (441 ± 121 vs. 378 ± 70, *p* = 0.034) levels on day 6 compared with the conventional treatment group. The changes in MMP-9 and VEGF were significant, starting on day 3 in the butylphthalide group but on day 6 in the conventional treatment group. There were no differences between the two groups in the NIHSS scores at admission and at discharge (*p* > 0.05). The overall response rate was higher in the butylphthalide group compared with the conventional treatment group (63.0 vs. 37.5%, *p* = 0.042).

**Conclusion:** Butylphthalide combined with conventional treatment can decrease MMP-9 levels and increase VEGF levels. The patients showed the reduced NIHSS scores, possibly suggesting some improvement in prognosis after stroke. Still, the conclusions need to be confirmed in a larger sample and in different etiological subtypes of stroke.

## Introduction

A stroke is an episode of acute neurological dysfunction from either ischemic infarction or a collection of blood within the brain or ventricular system with a resultant focal injury of the central nervous system (CNS) (1). Ischemic strokes (80–87% of strokes) result from cardioembolism (often from atrial fibrillation), large artery atherosclerosis (embolus or thrombosis), small vessel occlusion (lacunar), or systemic hypoperfusion (1). The estimated global incidence of stroke is 2–3 per 1,000 person-years, with older patients and patients with carotid artery stenosis or atrial fibrillation having the highest risk (1, 2). The mortality due to stroke every year in China accounts for nearly one-third of the total stroke-related deaths worldwide (3). Stroke disease is the main cause of non-traumatic disability and death in China (3). In Jiangxi Province, the incidence of cerebrovascular diseases is higher than in the other provinces of China, with ischemic stroke being predominant (about 73.5%) (4). The acute management of stroke includes blood pressure management, airway support, maintaining blood glucose levels, thrombolytics, endovascular therapy, aspirin, and decompressive surgery (2). In China, complementary traditional Chinese medicine (TCM) is also used for acute stroke management (5, 6).

Matrix metalloproteinase (MMP)-9, also called gelatinase B, is the MMP with the highest expression after hypoxic-ischemic changes and is closely related to the occurrence and development of cerebral infarction (7–10). Normally, its expression in the brain is low, and it is present in the form of an inactive zymogen (7–10). During the inflammatory responses of brain ischemia and hypoxia, the white blood cells, microglia, and astrocytes can produce MMP-9, and the MMP-9 levels are closely associated with the severity of cerebral infarction (7–10). Other studies have also shown that elevated MMP-9 levels are present both in the infarcted tissues and the tissues around the infarction, indicating that MMP-9 might play a role in the expansion of the infarct size (11–13).

The vascular endothelial growth factor (VEGF)-A is the most abundant member of the VEGF family, and it is involved in angiogenesis, promoting neovascularization (14). VEGF is involved in many processes such as atherosclerosis, cerebral edema, arteriogenesis, neuroprotection, neurogenesis, angiogenesis, brain, and vascular repair after ischemia (14–16). Normally, vascular endothelial cells, as the target cells of VEGF, cannot promote VEGF expression, but under pathological states, the concentration of VEGF can change rapidly, and increased VEGF levels are observed after stroke (17, 18).

Butylphthalide is a novel drug independently developed by China to treat cerebral infarction, which includes dl-3-n-butylphthalide (NBP) as the main active component (C_12_H_14_O_2_). It is a phthalide, an oily liquid with a taste of celery. It has the same structure as the natural substance levorotatory butylphthalide and is a synthetic racemic mixture. It is present in TCM ingredients such as chuanxiong (*Ligusticum chuanxiong hort*), Chinese lovage, and Chinese angelica, among others. Butylphthalide is an important candidate for the management of neurologic diseases by favoring the reconstruction of the microcirculation and protecting the mitochondrial functions (19, 20). Previous studies showed that butylphthalide improves the local microcirculation, increases blood perfusion, and increases the number of capillaries in ischemic brain areas (21–25). Butylphthalide also increases vasodilation and inhibits platelet aggregation (26). NBP can inhibit thrombus formation (27). One study showed that butylphthalide increases VEGF expression in animal models of stroke (24). Another animal study showed that butylphthalide decreases poststroke inflammation and MMP-9 expression (28). The effects of butylphthalide on VEGF and MMP-9 have to be confirmed in humans.

Therefore, this study aims to explore whether butylphthalide combined with conventional treatment can change the levels of MMP-9 and VEGF and the National Institutes of Health Stroke Scale (NIHSS) scores of patients with stroke and improve their prognosis. The results could provide data for the eventual inclusion of butylphthalide in routine stroke management.

## Methods

### Study Design and Participants

This was a prospective cohort study involving inpatients admitted to the Department of Neurology of Jiangxi Provincial People's Hospital from January to June 2019 due to acute cerebral infarction (stroke).

Stroke was diagnosed according to the criteria for acute ischemic stroke in the Chinese Guidelines for Diagnosis and Treatment of Acute Ischemic Stroke 2018 (29).

The inclusion criteria were: (1) 40–80 years of age; (2) meeting the TCM and Western diagnostic criteria for stroke; (3) cerebral infarction in the internal carotid artery system; (4) admitted to the hospital within 48 h of disease onset; and (5) new infarct lesion found by head MRI diffusion-weighted imaging. The exclusion criteria were: (1) cerebral infarction due to other causes after auxiliary examinations (such as tumors, parasitic infections, vascular malformations, and immune system diseases); (2) history of allergy to butylphthalide drugs or celery; (3) underwent thrombectomy after admission; (4) death within 72 h after admission; (5) liver and renal dysfunction; (6) pre-existing immune diseases, coagulation dysfunction, or bleeding tendency; (7) confirmed mental illness; or (8) women during pregnancy and lactation.

The patients were grouped according to the treatments they received. The patients who received butylphthalide and conventional treatment were assigned to the butylphthalide group, while all other patients were included in the conventional treatment group.

This study was approved by the Ethics Committee of Jiangxi Provincial People's Hospital (No. 20202). All the patients provided a written informed consent. The study was conducted according to the Declaration of Helsinki.

### Conventional Treatments

The therapeutic regimens for stroke were based on the Chinese Guidelines for Diagnosis and Treatment of Acute Ischemic Stroke 2018 (29). General treatments included maintenance of vital signs. The regulation of hypertensive symptoms was individualized. For patients with acute cerebral infarction within 24 h, antihypertensive drugs were administrated only when the systolic blood pressure was ≥200 mmHg or the diastolic blood pressure was >110 mm Hg. Within 24 h of the onset of cerebral infarction, the blood pressure of the patient was prevented from dropping by >15%. Patients with poststroke hypotension could receive expansion and elevation of blood pressure, as appropriate. Oxygen inhalation was performed if necessary to maintain the oxygen saturation at >94%. Patients with airway dysfunction underwent intubation, tracheotomy, auxiliary respirator, and other supporting treatments. After cerebral infarction, routine electrocardiogram and echocardiography were performed. If heart diseases such as atrial fibrillation or arrhythmia occurred, interventions were conducted to maintain a normal heart rate. For patients with cerebral infarction in the acute stage, drugs were used to regulate the blood glucose, so that the postprandial blood glucose was maintained at 7.8–10 mmol/l. Body temperature was controlled within the normal range, and if necessary, physical cooling, drug cooling, and other treatments were performed.

According to the onset time, the corresponding thrombolytic drugs were selected for eligible patients who were within the thrombolytic time window. For acute onset within 3–4.5 h, alteplase 0.9 mg/kg (including 10% intravenous injection) was intravenously administrated. Within 6 h of onset, intravenous infusion of 1–1.5 million units of urokinase was administrated for 30 min. According to the symptoms of the patient, the NIHSS score, and imaging results, aspirin 100 mg qd or clopidogrel 75 mg qd was administrated for antiplatelet therapy. If necessary, a loading dose or a combination of multiple antiplatelet drugs was given. Statins were given to patients with an atherosclerotic plaque to lower lipids and stabilize plaque. Neuroprotective drugs were used according to the conditions of the patient.

In addition, based on the disease severity, complications such as pneumonia, urinary tract infection, deep vein thrombosis, bedsores, and malnutrition were actively prevented using antibiotics, nasal feeding of liquid diet, urethral catheterization, and pressure stockings were performed when necessary. According to the conditions of the patient, limb functional exercise in moderation could be conducted to promote recovery.

### Butylphthalide Treatment

In addition to the conventional treatments, the patients in the butylphthalide group were treated with butylphthalide (NMPA approval number: H20100041; CSPC Enbipu Pharmaceutical Corporation Ltd., Shanghai, China) in sodium chloride for injection (butylphthalide 25 ml; sodium chloride 0.9 g/100 ml), bid, slowly dripped over at least 50 min. The treatment was maintained for 2 weeks, and then it was changed to oral administration. Butylphthalide soft capsule was orally administrated, 0.2, tid, for 20 days (NMPA approval number: H20050299; CSPC Enbipu Pharmaceutical Corporation Ltd., Shanghai, China).

### Baseline Data Collected Included

Baseline data, including age and sex, were collected at admission.

### Matrix Metalloproteinase-9 and VEGF

Plasma MMP-9 and VEGF levels were detected by double antibody sandwich ELISA. On days 1, 3, and 6 after admission, 2 ml of fasting venous blood was collected in EDTA tubes to measure the levels of MMP-9 and VEGF. The samples were centrifuged at 2–8°C and 1,000 × *g* for 15 min. After centrifugation, the plasma was separated as 1,000-μl aliquots in EP tubes and stored at −80°C. The samples were tested using the SEA553Hu 96T MMP9 detection kit (Cloud-Clone Corporation, Houston, Texas, USA) and the SEA553Hu 96T VEGF detection kit (Cloud-Clone Corporation, Houston, Texas, USA) and an iMark microplate reader (Bio-Rad, Hercules, California, USA).

### National Institutes of Health Stroke Scale

After admission, the NIHSS was used to evaluate the neurological deficits and disease severity (30) and on days 1 and 6 after admission. The changes in the NIHSS score were used to evaluate the effects of the two therapeutic regimens. For patients receiving thrombolytic therapy after admission, the NIHSS score on day 1 was the score immediately after thrombolysis. According to the Clinical Neurological Deficit Score for Stroke Patients jointly developed by the Chinese Neuroscience Society and Chinese Neurosurgery Society (31), and the degree of changes in the NIHSS score before and after treatment, the therapeutic effects were evaluated: (1) markedly effective: NIHSS score reduced by 91–100%; (2) effective: NIHSS score reduced by 46–90%; and (3) ineffective: NIHSS score reduced by <45% or increased. Overall response rate = markedly effective + effective rate.

### Statistical Analysis

All the statistical analyses were performed using SPSS 26.0 (IBM Corporation, Armonk, New York, USA). The Shapiro–Wilk normality test was conducted on all the continuous variables (Supplementary Table S1). Normally distributed data are presented as means ± SD and were analyzed using the Student's *t*-test. Non-normally distributed continuous variables are presented as medians [interquartile range (IQR)] and analyzed using the Mann–Whitney *U* test. The categorical data are presented as *n* (%) and were analyzed using the chi-squared test or the Fisher's exact test. The levels of MMP-9 and VEGF at 1, 3, and 6 days in each group were analyzed by repeated-measures the ANOVA and Tukey's *post hoc* test. All the statistical tests were two-tailed and *p* < 0.05 are considered as statistically significant.

## Results

### Characteristics of the Participants

**Figure 1:**
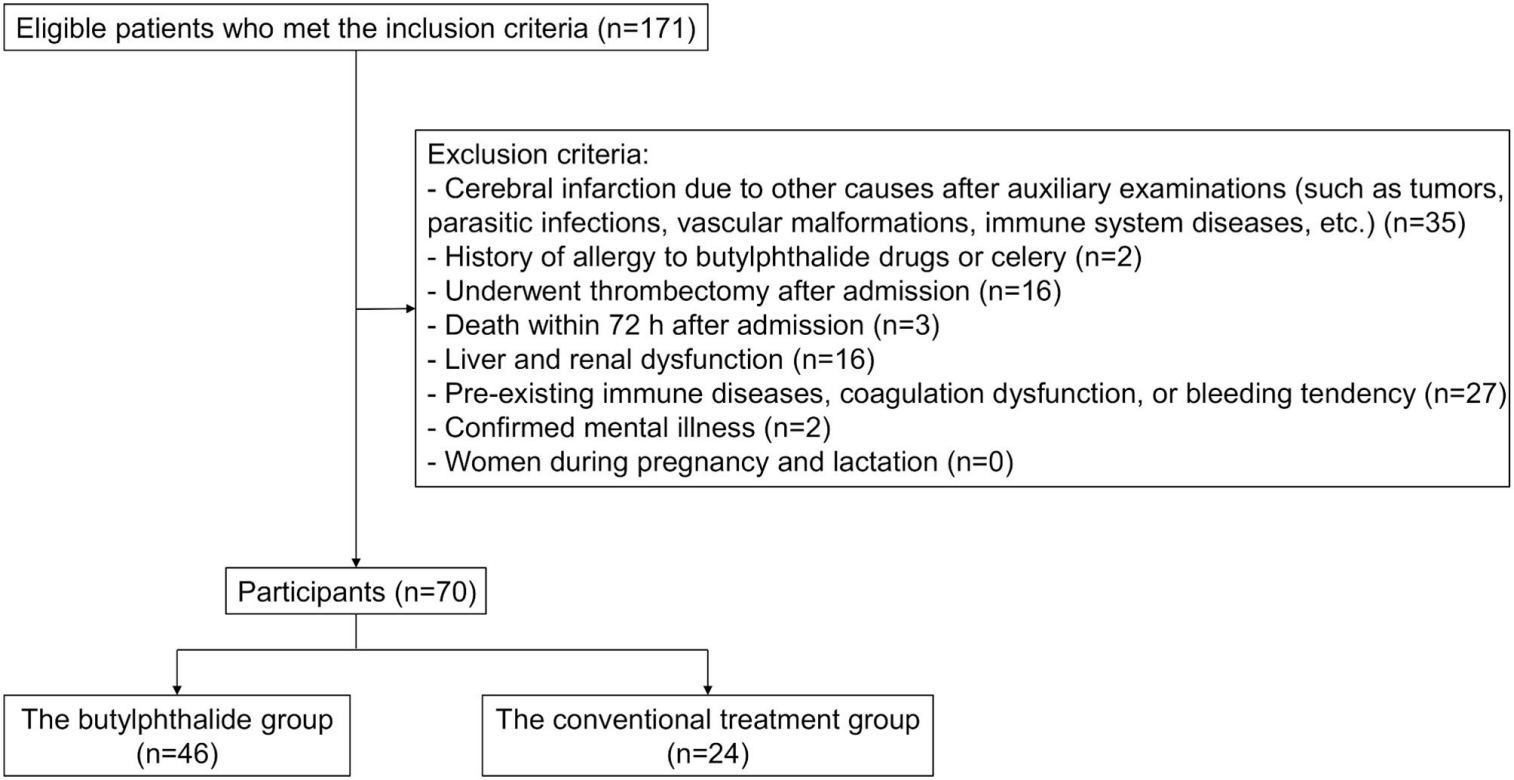
Consort figure.

During the study period, 171 patients were screened for eligibility. Among them, 35 were excluded due to cerebral infarction from other causes, two for a history of allergy to butylphthalide drugs or celery, 16 because they underwent thrombectomy after admission, three for death within 72 h after admission, 16 for liver and renal dysfunction, 27 for pre-existing immune diseases, coagulation dysfunction, or bleeding tendency, and two for confirmed mental illness. Hence, 70 patients were included in the analysis: 24 in the conventional treatment group and 46 in the butylphthalide group (Figure 1). Table 1 presents the characteristics of the participants.

**Table 1:** Characteristics of the participants.

	**Butylphthalide group** **(*n* = 46)**	**Conventional treatment group** **(*n* = 24)**	***p*-value**
Age (years)	64.6 ± 8.9	63.0 ± 1.0	0.481
Sex, male, *n* (%)	28 (61%)	15 (63%)	0.894

### Matrix Metalloproteinase-9 Levels

**Figure 2:**
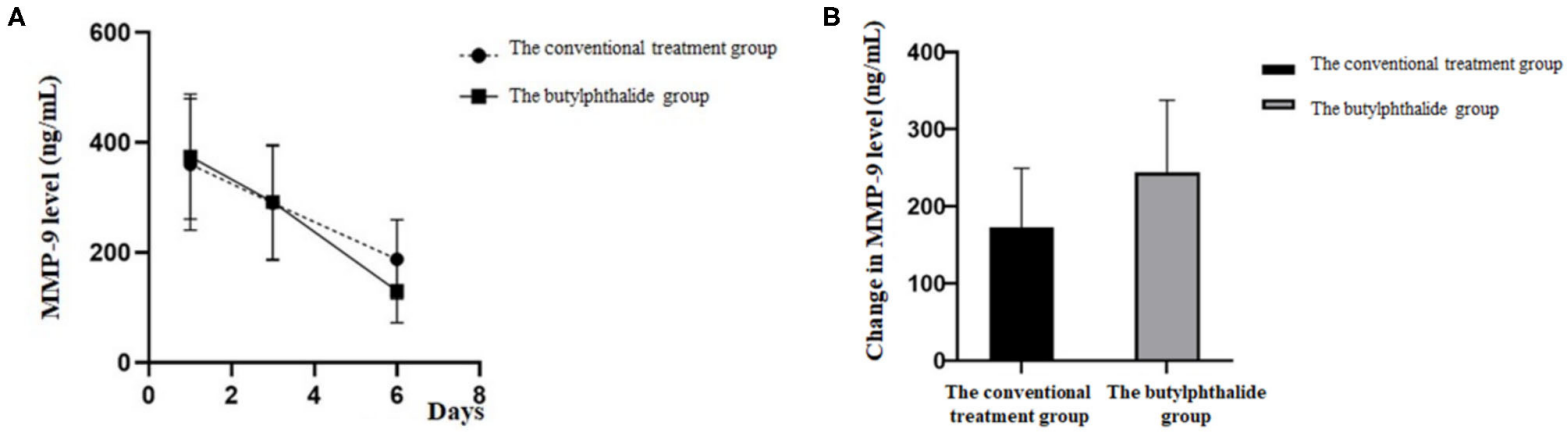
Matrix metalloproteinase-9 (MMP-9) level on the first, third, and sixth day of treatment **(A)** and the change in MMP-9 level between the two groups **(B)**.

Table 2 shows the MMP-9 levels in the two groups. There were no differences in MMP-9 levels between the two groups on days 1 and 3, but the butylphthalide group showed lower MMP-9 levels on day 6 compared with the conventional treatment groups (130.18 ± 58.84 vs. 187.71 ± 65.25, *p* = 0.001), and greater changes from day 1 to day 6 (243.84 ± 94.05 vs. 172.39 ± 77.01, *p* = 0.002). In the butylphthalide group, the MMP-9 steadily decreased (all *p* < 0.05), while in the conventional treatment group, the change from day 1 to day 3 was not significant (*p* = 0.086) (Figure 2).

**Table 2:** Changes in matrix metalloproteinase-9 (MMP-9) after admission according to the treatment groups.

**MMP-9**	**Butylphthalide group** **(*n* = 46)**	**Conventional treatment group** **(*n* = 24)**	***p*-value**
Day 1	374.02 ± 115.12	360.10 ± 121.99	0.639
Day 3	291.84 ± 105.47	289.96 ± 91.68	0.941
Day 6	111.85 (89.51, 174.83)	187.71 ± 65.25	0.001
Δ Day 1–Day 6	243.84 ± 94.05	172.39 ± 77.01	0.002
Day 1 vs. 3	0.02	0.086	/
Day 3 vs. 6	<0.001	<0.001	/
Day 1 vs. 6	<0.001	<0.001	/

**Figure 3:**
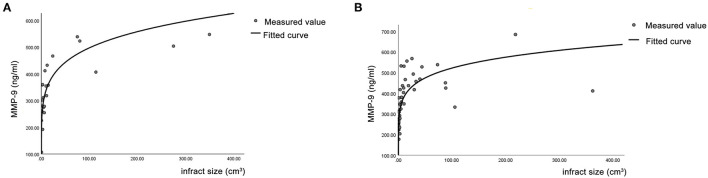
Correlation analysis between the infarct volume and MMP-9 (day 1). **(A)** The conventional treatment group. **(B)** The butylphthalide group.

Correlation analysis showed that the larger the infarct volume, the higher the concentration of MMP-9 (day 1), and the change was significantly correlated in both butylphthalide (*Y* = 275.72 × *X*^0.14^, *R*^2^ = 0.578, *p* = 0.005) and conventional treatment (*Y* = 231.61 × *X*^0.17^, *R*^2^ = 0.667, *p* = 0.007) groups (Figure 3).

### Vascular Endothelial Growth Factor Levels

**Figure 4:**
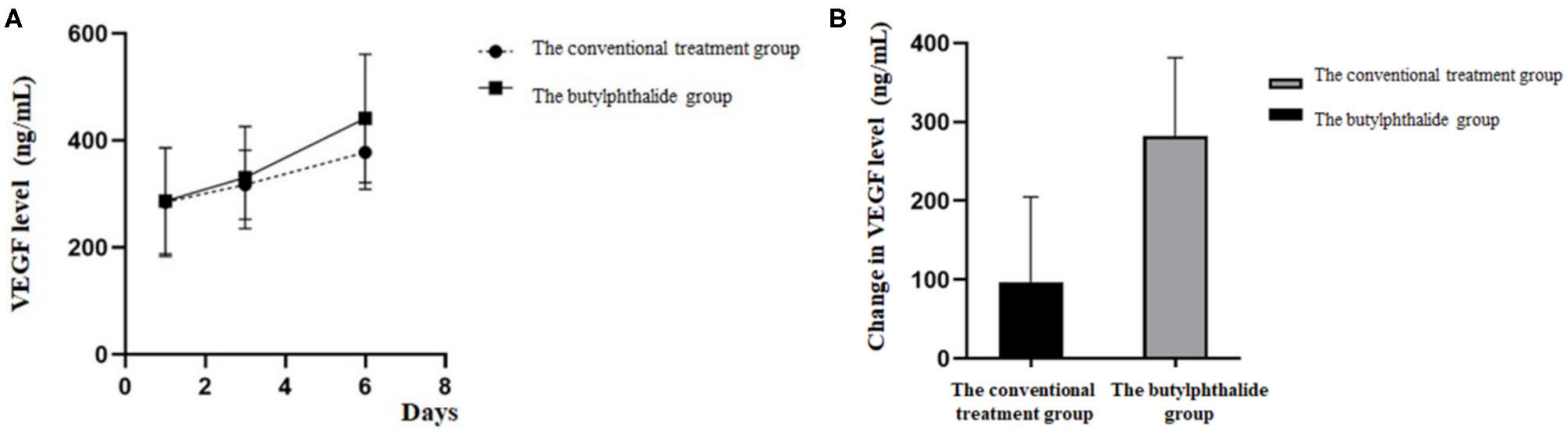
Vascular endothelial growth factor (VEGF) level on the first, third, and sixth day of treatment **(A)** and the change in VEGF level between the two groups **(B)**.

Table 3 shows the VEGF levels in the two groups. There were no differences in VEGF levels between the two groups on days 1 and 3, but the butylphthalide group showed higher VEGF levels on day 6 compared with the conventional treatment groups [415.60 (346.39, 505.22) vs. 380.01 (329.36, 441.43), *p* = 0.034] and greater changes from day 1 to day 6 [145.86 (92.99, 211.16) vs. 127.14 (29.53, 169.97), *p* = 0.018]. In the butylphthalide group, the VEGF steadily increased (*p* < 0.05), while in the conventional treatment group, the change from day 1 to day 3 was not significant (*p* = 0.097) (Figure 4).

**Table 3:** Changes in vascular endothelial growth factor (VEGF) after admission according to the treatment groups.

**VEGF**	**Butylphthalide group** **(*n* = 46)**	**Conventional treatment group** **(*n* = 24)**	***p*-value**
Day 1	259.72 (208.51, 340.06)	255.24 (216.56, 360.95)	0.951
Day 3	309.04 (255.12, 405.28)	327.44 (265.65, 377.26)	0.931
Day 6	415.60 (346.39, 505.22)	380.01 (329.36, 441.43)	0.034
Δ Day 6–Day 1	145.86 (92.99, 211.16)	127.14 (29.53, 169.97)	0.018
Day 1 vs. 3	0.01	0.097	/
Day 3 vs. 6	<0.001	<0.001	/
Day 1 vs. 6	<0.001	0.001	/

### National Institutes of Health Stroke Scale Scores

**Figure 5:**
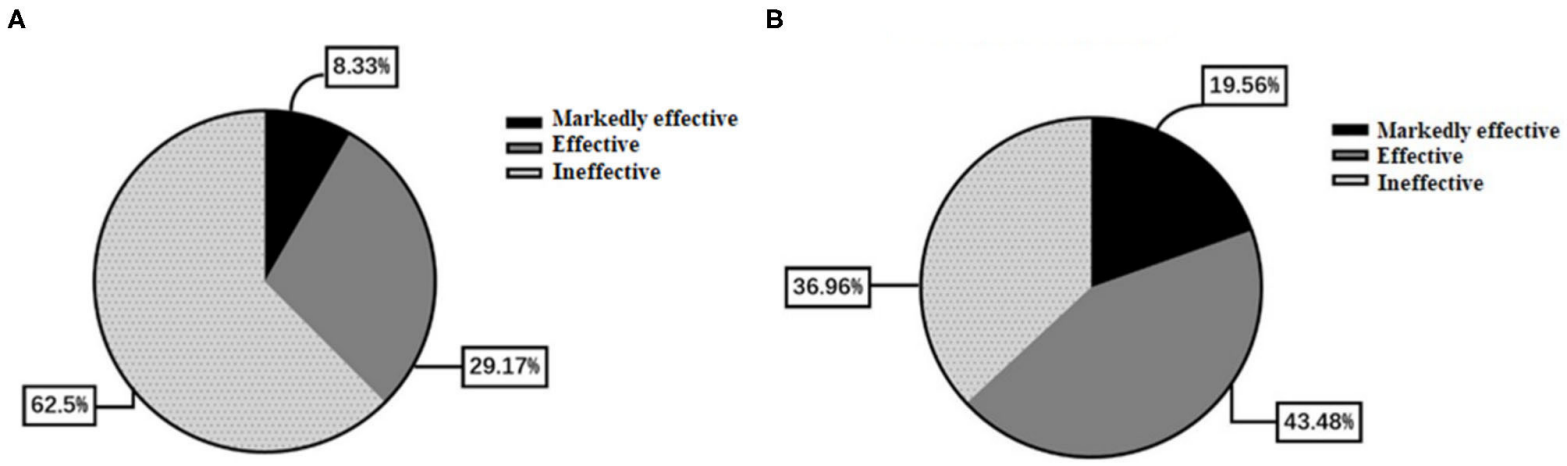
Comparison of effectiveness between the two groups. **(A)** The conventional treatment group. **(B)** The butylphthalide group.

Table 4 presents the NIHSS scores in the two groups. There were no differences between the two groups in the NIHSS scores at admission and at discharge (all *p* > 0.05). The overall response rate was higher in the butylphthalide group compared with the conventional treatment group (63.0% vs. 37.5%, *p* = 0.042) (Figure 5).

**Table 4:** Changes in the National Institutes of Health Stroke Scale (NIHSS) after admission according to the treatment groups.

**NIHSS**	**Butylphthalide group** **(*n* = 46)**	**Conventional treatment group** **(*n* = 24)**	***p*-value**
Score on admission	5.0 (2.8, 13.3)	5.5 (2.0, 10.0)	0.765
Score on discharge	4.0 (1.0, 7.8)	4.0 (1.0, 7.8)	0.492
Score difference	2.0 (1.0, 4.3)	1.5 (1.0, 3.5)	0.361
Markedly effective	9	2	/
Effective	20	7	/
Ineffective	17	15	/
Overall response rate	63.00%	37.50%	0.042

## Discussion

Butylphtalide increases the VEGF and MMP-9 in animal models of stroke (24, 28) and might be used as key biomarkers in the management of stroke, but data in humans are lacking. This study aimed to explore whether butylphthalide combined with conventional treatment can change the levels of MMP-9 and VEGF and the NIHSS scores of patients with stroke. The results strongly suggest that butylphthalide combined with conventional treatment can decrease MMP-9 levels, increase VEGF levels, and reduce the NIHSS scores, possibly improving the prognosis after stroke.

This study showed that butylphthalide could decrease the MMP-9 levels after stroke more effectively than conventional treatments alone. Such an effect of butylphthalide was observed in rat and mouse models of stroke (28, 32), but this effect was not observed in models of chronic cerebral hypoperfusion (33), probably because MMP-9 is mainly expressed in the acute phase of the stroke. MMP-9 expression in the brain after infarction is directly related to the infarct size and brain injury (7–10). MMPs promote blood-brain barrier permeability, edema, and hemorrhagic transformation (34, 35). Hence, decreasing the MMP-9 levels promptly after stroke onset is conducive to limiting the extent of brain injury (11–13).

Increased expression of VEGF after stroke is a protective factor since it participates in neovasculogenesis, which increases brain perfusion and oxygenation (17, 18). This study showed that butylphthalide increased the VEGF levels to a greater extent and quicker than conventional treatments alone. This is supported by animal studies (24, 36, 37) and by one study in humans (38).

Still, the exact relationship between MMP-9 and VEGF is unclear. High levels of MMP-9 in models of retinopathy upregulate the expression of VEGF to promote retinal neovascularization (39). Indeed, MMPs can process matrix-bound VEGF and increase its bioavailability (40). Still, since MMP-9 levels were decreased but VEGF was increased, it is possible that influences MMP-9 and VEGF levels that are independent of the effect of MMP-9 on matrix-bound VEGF.

Animal studies showed that butylphthalide increases the local microcirculation (4, 7, 8, 14, 18), has vasodilation effects, and inhibits platelet aggregation (2). Therefore, butylphthalide might improve brain blood perfusion after stroke. The changes in MMP-9 and VEGF induced by butylphthalide possibly participated in the higher objective response rate observed in the butylphthalide group. Such improvements were also observed in animal studies (24, 28, 32, 36, 37). Butylphthalide also possesses antiplatelet aggregation and antithrombotic effects, protects the mitochondrial functions, prevents neuronal apoptosis, reduces oxidative stress, and improves neurogenesis, which all participate in better outcomes after stroke (20, 41). Jia et al. (42) showed that butylphthalide improves the outcomes of patients with subcortical vascular cognitive impairment without dementia. Qian et al. (43) showed that butylphthalide combined with kallidinogenase or edaravone enhanced the independence rate of patients with ischemic stroke. Future studies should examine those effects of butylphthalide in humans.

This study has some limitations. The sample size was small and from a single center. There was no randomization, and butylphthalide was given according to the judgment of the physician, which probably introduced biases. The VEGF and MMP-9 levels were detected at only three time points. Follow-up and chronic-phase data are not available. Only MMP-9 and VEGF were examined, and many other proteins involved in brain damage modulation after stroke are probably also affected by butylphthalide. No follow-up was performed after discharge, and the mid- and long-term outcomes of the patients are unknown. At last, the NIHSS scores of patients with mild stroke may be subjectively affected. Additional studies are necessary to address those issues. A randomized trial is currently underway (ClinicalTrial.gov NCT03539445).

In conclusion, butylphthalide combined with conventional treatment can decrease MMP-9 levels and increase VEGF levels. The patients showed reduced NIHSS scores, possibly suggesting some improvement in prognosis after stroke. Still, the conclusions need to be confirmed in a larger sample and in different etiological subtypes of stroke.

## Data Availability Statement

The original contributions presented in the study are included in the article/Supplementary Material, further inquiries can be directed to the corresponding author/s.

## Ethics Statement

The studies involving human participants were reviewed and approved by the Ethics Committee of Jiangxi Provincial People's Hospital (No. [2020]2). The patients/participants provided their written informed consent to participate in this study.

## Author Contributions

YXi and YXu carried out the studies, participated in collecting data, and drafted the manuscript. JL and SC performed the statistical analysis and participated in its design. SX and XZ helped to draft the manuscript. All the authors read and approved the final manuscript.

## Funding

This study was supported by the Science and Technology Project of the Health Commission of Jiangxi Province (Grant Number 20195025). The funders had no role in study design, data collection, and analysis, decision to publish, or preparation of the manuscript.

## Conflict of Interest

The authors declare that the research was conducted in the absence of any commercial or financial relationships that could be construed as a potential conflict of interest.

## Publisher's Note

All claims expressed in this article are solely those of the authors and do not necessarily represent those of their affiliated organizations, or those of the publisher, the editors and the reviewers. Any product that may be evaluated in this article, or claim that may be made by its manufacturer, is not guaranteed or endorsed by the publisher.
