# Supplementary material for: Butylphthalide Combined With Conventional Treatment Attenuates MMP-9 Levels and Increases VEGF Levels in Patients With Stroke: A Prospective Cohort Study
Source: Front Neurol. 2021 Dec 20;12:686199. doi: 10.3389/fneur.2021.686199 (PMC8720749; doi:10.3389/fneur.2021.686199)
Supplement: Supplementary file 1 [file Table_1.docx]

**Supplementary Table S1.** Results of the normality tests

|  | ***p* value** |
| --- | --- |
| **Butylphthalide group** |  |
| Age | 0.097 |
| MMP-9 (day 1) | 0.333 |
| MMP-9 (day 3) | 0.535 |
| MMP-9 (day 6) | < 0.001 |
| MMP-9 (day 6-day 1) | 0.409 |
| VEGF (day 1) | 0.005 |
| VEGF (day 3) | 0.018 |
| VEGF (day 6) | 0.016 |
| VEGF (day 6-day 1) | 0.448 |
| NIHSS (admission) | < 0.001 |
| NIHSS (discharge) | < 0.001 |
| NIHSS (discharge-admission) | < 0.001 |
| **Conventional treatment group** |  |
| Age | 0.461 |
| MMP-9 (day 1) | 0.681 |
| MMP-9 (day 3) | 0.335 |
| MMP-9 (day 6) | 0.743 |
| MMP-9 (day 6-day 1) | 0.599 |
| VEGF (day 1) | 0.186 |
| VEGF (day 3) | 0.404 |
| VEGF (day 6) | 0.570 |
| VEGF (day 6-day 1) | 0.066 |
| NIHSS (admission) | 0.004 |
| NIHSS (discharge) | < 0.001 |
| NIHSS (discharge-admission) | < 0.001 |
